# Restless leg syndrome a common undiagnosed comorbidity of clinical significance in cirrhosis 

**Published:** 2019

**Authors:** Akash Rajender, Saumya Mathur, Priyanka Choudhary, Shalini Upadhyay, Gaurav Rajender, Rajat Bhargava, Subhash Nepalia

**Affiliations:** 1 *Department of Gastroenterology, Mahatma Gandhi Medical College & Hospital, Jaipur, Rajasthan, India *; 2 *Department of General Medicine, Mahatma Gandhi Medical College & Hospital, Jaipur, Rajasthan, India*; 3 *Department of Psychiatry, SMS Medical College, Jaipur, Rajasthan, India *

**Keywords:** Restless leg syndrome, Cirrhosis, Child Turcotte Pugh

## Abstract

**Aim:**

Our aim was to study the prevalence and association of restless leg syndrome (RLS) in liver cirrhosis subjects.

**Background:**

Sleep disturbances are common in cirrhosis. RLS is a chronic sensorimotor sleep disorder with an irresistible urge to move limbs.

**Methods:**

Two hundred consecutive cirrhosis patients, presenting at Mahatma Gandhi Medical College & Hospital, Jaipur were evaluated. 157 subjects meeting the inclusion criteria were selected for further evaluation. Revised International Restless Legs Syndrome Study Group (IRLSSG) criteria 2012, was used for diagnosed of RLS. Severity of RLS was evaluated by a validated Hindi translation of International RLS severity (IRLS) scoring system.

**Results:**

Of studied 157 cirrhotic, the mean age was 46.4 ± 10 years, 109 (69.43%) males and 48 (30.57%) females. 41 (26.11%) cirrhotic subjects had RLS. Child Turcotte Pugh (CTP) Class (A 9/55, B 15/68, C 17/34; p 0.043) and alcohol as etiology for liver dysfunction (p 0.03) were significantly associated with RLS.

**Conclusion:**

RLS is common comorbidity in cirrhosis, especially in alcohol related cirrhosis. RLS has a clinical correlation with prognosis & severity of cirrhosis.

## Introduction

 Restless leg syndrome (RLS) or Ekbom’s syndrome (1), is a sleep disorder, with sensorimotor neurological symptoms. This includes an urge to move legs or other extremities during rest. This may be associated with unpleasant sensations relieved by movement. Its prevalence in general population is between 2.5 to 15 percent (2), prevalence increases with age and is higher among females (3). The revised international restless legs syndrome study group (IRLSSG) 2012, has described 5 essential clinical features to establish the diagnosis of RLS; (i) an urge to move the legs, usually but not always accompanied or caused by uncomfortable and unpleasant sensations in the legs; (ii) these symptoms begin or worsen during periods of rest or inactivity such as lying or sitting; (iii) are partially or totally relieved by movement; and (iv) symptoms are worse in the evening or nighttime; (v) not solely accounted by other medical or behavior condition. 

It is often overlooked as a relatively benign insignificant condition, but recent literature suggests it to be an important determinant of Quality of Life (QOL) (3). While several clinical conditions like iron deficiency (4), polyneuropathy (5), chronic kidney disease (6), and diabetes mellitus (7) have been associated with RLS, but there is still no significant literature investigating the association between cirrhosis and RLS. Since cirrhosis has been associated with sleep disturbance (8, 9) it becomes intriguing to evaluate its association with RLS. Impetus is also gained from recent works reported in Japan (10) & USA (11) by Matsuzaki T *et al.* & Franco RA *et al.* respectively. 

## Methods

In a prospective observational study, two hundred consecutive cirrhosis patients, presenting at Mahatma Gandhi Medical College & Hospital, Jaipur were evaluated from September 2016 to December 2017. A formal written consent was taken from all patients. Cirrhosis was diagnosed by clinical, radiology, laboratory and endoscopic features. Subjects younger than 18 years of age, severe anemia (Hemoglobin <7 g/dL), significant renal dysfunction (Serum creatinine >2 and/or clinical/radiologic evidence of chronic kidney disease), Grade 3/4 hepatic encephalopathy, drugs associated with RLS (dopaminergic agonist, anticonvulsants, antidepressant & neuroleptics) and clinically unstable subjects were excluded from the study. Also, pregnant women were excluded from the study, because of expected high prevalence of RLS, which might act as a confounding factor. 157 patients were selected after the initial screening for further evaluation. After collection of socio-demographic data, clinical and laboratory profiling of cirrhotic subjects was done. Revised International Restless Legs Syndrome Study Group (IRLSSG) 2012 was used for diagnosis of RLS in cirrhotic subjects. Subjects meeting the five criteria’s, with symptom frequency of at least two times per week were diagnosed as RLS.

In subjects with RLS, further severity of RLS was evaluated by a validated Hindi translation of International RLS severity (IRLS) scoring system ^12 ^which consisted of a set of 10 questions, each scored on a scale of 0 to 4. The maximum obtainable score being 40, RLS severity was classified as mild (0–10), moderate (11–20), severe (21–30), and very severe (31–40). Statistical analysis was carried out using the SPSS 12.0 software (SPSS, Inc., Chicago). A p value <0.05 was considered statistically significant. 

## Results

In our study we examined 157 cirrhotic subjects at Mahatma Gandhi Medical College, Jaipur. The mean age was 46.4 ± 10 years, 109 (69.43%) were males and 48 (30.57%) females. They were examined using clinical, laboratory & radiological variables. The etiology for cirrhosis (Table1) for majority of subjects was alcohol (58.6%) and hepatitis B (HBV) infection in 17.2% subjects. 49 (31.21%) met the IRLSSG 2012 criteria, on further evaluation of these subjects by a neurologist 41 (26.11%) were confirmed to suffer from RLS. The laboratory profile of evaluated cirrhotic subjects is shown in Table 2. Severity & prognosis of liver disease as determined by Child Turcotte Pugh (CTP) class (A: 9/55, B: 15/68, C: 17/34; p: 0.043) and alcohol as etiology for liver dysfunction (p: 0.03) was significantly associated with RLS (Table 1 & 3). Other etiologies of cirrhosis (HBV, HCV, autoimmune, NASH, and cryptogenic) showed comparable distribution of RLS, but less than alcoholics. Most cirrhosis patients diagnosed with RLS had moderately severe RLS (14, 34.15%) followed by severe (12, 29.27%).

**Table 1:** Etiological profile of cirrhotics

**Etiology**	**Cirrhotic (n=157)**	**RLS present (n=41)**	**RLS absent (n=116)**	**p value**
Alcohol	92(58.6%)	27 (65.85%)	65 (56.03%)	0.03*
HBV	27(17.2%)	9 (21.95%)	18 (15.52%)	0.23
HCV	5(3.18)	2 (4.88%)	3 (2.59%)	0.29
Autoimmune	1(0.64%)	0 (0.0%)	1 (0.86%)	0.3
NASH	3(1.91%)	1 (2.44%)	2 (1.72%)	0.3
Cryptogenic	29(18.47%)	2 (4.88%)	27 (23.27%)	0.2

* p Value <0.5, HBV-Hepatitis B virus, HCV-Hepatitis C Virus, NASH- Non Alcoholic Steatohepatitis, RLS- Restless Leg Syndrome

**Table 2:** Laboratory Profile of Cirrhotic patients

**Characteristic**	**Value (95% confidence interval)**
Haemoglobin (g/dL)	10.2 (5.4-16.2)
TLC (x 1000/µL)	6.6 (1.8-17.0)
Platelets (x 1000/µL)	92 (18-236)
Serum bilirubin (mg/dL)	1.6 (1.1-11.4)
Alanine aminotransferase (IU/L)	66 (22-351)
Aspartate aminotransferase (IU/L)	78 (17-628)
International Normalized ratio (INR)	1.4 (1.0-2.6)
Serum protein (g/dL)	6.1 (2.6-8.8)
Serum albumin (g/dL)	3.1 (1.4-4.2)
Serum creatinine	1.0 (0.5-2.8)

Data is represented as median (range)

**Table 3:** Correlation of Child Turcotte Pugh Class with Restless Leg Syndrome (RLS)

**CTP** * ** CLASS**	**N=157**	**RLS (N-=41)**	**NO RLS (N=116)**	**p Value**
A	55 (35.03%)	9 (21.95%)	44 (37.93%)	0.043†
B	68 (43.31%)	15 (36.59%)	54 (46.55%)	
C	34 (21.66%)	17 (41.46%)	18 (15.52%)	

* Child Turcotte Pugh;

† p<0.05

## Discussion

In this study we determined the prevalence of RLS in liver cirrhosis patients, its correlation with etiology of cirrhosis and prognostic marker. 41 (26.11%) cirrhotic subjects were found to suffer from RLS. RLS was significantly more common in subjects with alcoholic cirrhosis and subjects had higher CTP scores.

RLS is a type of sleep related movement disorder that affects approximately 10% population based on epidemiological surveys (12-16). It is a sensori-motor chronic neurological disorder, which is usually remains undiagnosed. Literature remains scarce on prevalence of RLS in Indian population. The only previous population based Indian study from Bangalore included 1266 subjects, suggested the prevalence of RLS as 2.1%, which is very low (17). There are very few studies worldwide assessing the prevalence of RLS in cirrhosis. Franco RA *et al*. (11) for the first time in 2008, reported a prevalence of 62% in USA using telephonic surveys. Following which Matsuzaki T *et al*. (10) in 2012 from Japan, suggested the prevalence of RLS in cirrhotic subjects as 16.8%. Iron deficiency has known association with RLS (18). Also iron deficiency is extremely common in cirrhotic patients owing to decreased intake, improper absorption, persistent occult blood loss, episodes of gastrointestinal bleed and decreased iron absorption for reduced gastric acidity. Poor nutrition status in Indian population and high prevalence of iron deficiency may predispose Indian cirrhotic patients for a higher prevalence of RLS as compared to their Japanese counterparts. As compared to telephonic surveys used in a previous study suggesting high prevalence, our study included structured evaluation, strict exclusion protocols, which may account for lower reported prevalence in our study.

Various mechanisms have been postulated for excessive risk of RLS in cirrhotic patients apart from iron deficiency. Decreased serotoninergic transmission caused by an excess of 5-HIAA (5-hydroxyindoleacetic acid) and blockade of serotonin release are evident in cirrhosis (19). Increased serotonin precursors such as tryptamine, quinolinic acid and tryptophan metabolites have been reported in brain and CSF of patients with liver disease (20, 21).

Cortico-spinal tract dysfunction has been reported in cirrhosis using magnetic resonance imaging (MRI). Fast Flair techniques in MRI have revealed reversible cortico-spinal tract abnormalities after transplantation. 20 RLS developing in diseases affecting cortico-spinal tracts is well described (22). Alcoholism, viral hepatitis, hemochromatosis, known etiologies of liver dysfunction can contribute or cause peripheral nerve disease/dysfunction (23). More advanced cirrhosis (CTP class C) and alcoholic etiology was more predisposed to develop RLS, probably owing to more severe neurological dysfunction, associated with prolonged underlying liver disease.

Our study had few limitations like, inclusion of subjects with known causes of neuropathy (eg diabetes, alcohol, renal dysfunction etc.), inability to confirm neuropathy using Nerve Conduction studies and inclusion of subjects with known risk factors of RLS (Small Intestinal Bacterial Overgrowth, inflammatory bowel disease, anemia, diabetes). Multicenter large longitudinal studies are needed to evaluate the prognostic relevance of RLS in cirrhosis, its clinical course, nature and effect of its treatment on various parameters.

## Conflict of interests

The authors declare that they have no conflict of interest.
